# Pay Hospitals in England

**Published:** 1900-11-03

**Authors:** 


### The Institutional Workshop.

### PAY HOSPITALS IN ENGLAND.

For twenty years public opinion in England has been shaping itself more and more in favour of some adequate provision in large towns for the accommodation of members of the middle and lower middle class especially who can afford to pay about three guineas a week inclusive for the accommodation and treatment they need when ill. In all the more civilised countries in Europe and in the United States accommodation of this kind is more or less readily obtainable at even lower rates than those mentioned. Indeed the Continental system is based upon the sound principle that everybody who requires hospital care should pay something for that care, and this method is in force in various countries, including Norway, Sweden, and Denmark, where the patients are grouped in divisions, say, one to four, each class getting the accommodation which is suitable to the weekly charge made. The lowest class—class 4—on this plan is paid for by the municipality or poor-law authorities, and the payments for the other three classes are made by the patients or their friends. In England every year more and more patients resort to the hospitals, because the hospitals each year become more and more efficient as institutions where disease is treated with increasing success under the most favourable conditions which modern science and medical skill can provide. It follows that in England the doctors on the one hand not unnaturally complain that the continuous increase in the number of hospital patients renders it more and more difficult for the general practitioners to get a living, and on the other that our present system of hospital relief in effect tends to pauperise rather than to raise the standard of independence amongst the people generally. Yet each attempt which has been made to introduce paying wards for poor paying patients into general hospitals in England has been looked at askance by the medical profession for the most part, and is not popular with subscribers to hospitals or even with the central funds who distribute money amongst hospitals, although the need for such accommodation is generally admitted, and its efficacy, if the administration of our hospitals were sound, can be conclusively proved as a remedy for reducing very largely the risks arising from the present almost universal system of eleemosynary hospital treatment.

### Private Institutions or Homes of Treatment.

About twenty-three years ago a movement was started, under the presidency of the late Duke of Northumberland, whereby a few relatively wealthy people subscribed about £25,000 and incorporated themselves under license by the Board of Trade as the Home Hospitals Association for Paying Patients. The object of that Association was to set up a pay hospital which should be replete with every modern sanitary arrangement, and to provide accommodation at a remunerative rate for those members of the population who were willing to pay for it, and who had to go to London to be operated upon by eminent surgeons, or to receive treatment at the hands of consultants resident in London. At that time practically the only accommodation for such cases was to be found in indifferent lodgings in the smaller streets of the West End, which were often insanitary and almost invariably uncomfortable and ill adapted to the purpose. The Home Hospitals Association bought, reconstructed, and fitted up Nos. 16 and 17 Fitzroy Square, which have ever since been occupied by cases undergoing operation and treatment, and which have been conducted successfully at a small profit. To this day Fitzroy House, the home hospital in question, provides good accommodation at relatively lower rates than obtained elsewhere for identical accommodation. The immediate object of the founders of the Home Hospitals Association was to provide suitable hospital accommodation for the middle classes for payment; but they hoped that when it was proved that a hospital could be fitted up and maintained at a profit, the managers of the general hospitals would realise that they could properly meet the requirements of the increasing population of a great city like London by opening paying wards to which they would admit patients for an inclusive payment of from two guineas a week upwards. This object has not yet been fulfilled, mainly because hospitals like St. Thomas's, Guy's, and the Great Northern Central, where this system has been introduced, have been attacked for providing the accommodation in question on the ground mainly that these paying wards were subject to as much abuse as the free beds, because they were utilised by a class of the community who would afford to pay, and ought to pay, a much higher rate than that charged for their accommodation when sick.

There has been the further and sounder objection that in opening these paying wards the authorities of the general hospitals in question made no provision for the payment of the medical men who were charged with the treatment of these paying patients. For upwards of twenty years the Home Hospital in Fitzroy Square has been utilised by a large number of leading consultants in London for their private patients without any difficulty arising on this latter score because the rules provide that each medical man shall make his own arrangements with the patients under his care with regard to his own remuneration. It is contended, however, that any such system, if followed by the general hospitals in regard to their paying wards, would break down because the patients could not afford to pay an adequate fee, and there would be great objection on the part of the staff, as well as administrative difficulties, if the private medical attendant of a patient were to follow him for purposes of treatment to the paying ward of a general hospital. We have pointed out in these columns over and over again—and it is a demonstrable fact—that in the case of a hospital like the Great Northern Central, where the paying wing was specially designed for the purpose of accommodating pay patients, where the medical attendant can enter and leave the paying ward without entering the general hospital, no difficulty of the kind should be possible, providing the authorities funded a certain percentage of the payments made by each patient out of which the fees of the medical attendant would be paid. In this way each patient could readily have the services of a medical practitioner of his own selection at a fee within the patient's means, and no administrative difficulties of any kind could arise where the administration of the hospital was efficient. Every general hospital in England, in fact, which will lay itself out to provide a separate wing, distinct from the general hospital, for the accommodation of paying patients, and will adopt the funding system of payment for the medical treatment of the cases received into that wing, can add enormously to its usefulness by providing the poor paying patients, for a reasonable payment, in the district which it serves with the hospital treatment and accommodation which they may need in the day of sickness or accident.

### Schemes in the Air.

The general hospitals have not as yet seen their way, as we have already stated, to take up this question. They find that, although they get more and more patients every year, they also receive more and more contributions from the public, and so the abuses which existed twenty years ago tend to increase rather than to diminish, because the hospitals are deservedly more popular than ever they were before in the history of voluntary institutions. Besides in these days of education every wise person when ill seeks relief at the source which is known to provide treatment of the very highest kind whereby recovery is most likely to be expedited and secured.

Failing any step on the part of the general hospitals, private individuals, some of whom are wealthy, are at the present time wishful to expend many thousands of pounds in the erection of a pay hospital, distinct and under separate management, for the accommodation of the poorer paying patients. It is undoubtedly true that if such an institution were to be erected in the northern, southern, eastern, and western districts of London its wards would be full, and under proper management there can be no doubt that these institutions would fulfil a very useful purpose. On the plan indicated above, which provides for the treatment of each case by the practitioner selected by the patient, who would be paid out of the fund set apart for that purpose, there should be no administrative difficulty, and these establishments would make it possible to bring pressure to bear on the general hospitals to be more careful in the selection of cases admitted to their wards without payment of any kind. We are strongly of opinion that if one such institution was opened and administered upon the right lines, others would without doubt follow, to the great benefit of large numbers of the community who at present suffer unnecessary hardships when ill. There is, we understand, another and a larger scheme at the present time under consideration whereby capitalists propose to invest a considerable sum of money in the erection of a large building for the reception of patients who can pay from three to four guineas a week, where they can be attended by their own doctors, and where they would have every accommodation provided for them during their residence as patients. The plan favoured is said to be that adopted in Vienna, where the institution in question is in reality a large hotel for sick people; and we are inclined to think that any such scheme, if carried out in London, however popular it might be from the patients' point of view, would find itself in financial difficulties if the scheme were based upon ordinary business lines, and the capital was provided with the idea of yielding an adequate dividend. This opinion is based upon the knowledge that, since the opening of Fitzroy House as a home hospital twenty years ago, at least fifty nursing homes of sorts, from the most efficient to the relatively inefficient, have been opened in London where patients are received. The history of these homes, with very few exceptions, has not been encouraging financially, and, as far as our inquiries go, we are led to the conclusion that the profits on the whole are so limited as to be inadequate to satisfy anyone but a woman, who, with the devotion of her sex, plods on under many discouragements, content with an annual income which, having regard to the risks run and the expenditure incurred, any business man would consider to be quite inadequate as a return upon the capital invested.

We shall be interested to watch the further developments of the two larger schemes which are now in the air, and we have thought it well to call attention to this matter at the moment because we are confident that it is well worth the consideration of the committee of every important hospital which has land available whereon it could erect a suitable paying wing for the accommodation of poorer paying patients under regulations which would satisfy the requirements of the public and secure the support of the medical profession.

